# Consensus guideline for the diagnosis and treatment of aromatic l-amino acid decarboxylase (AADC) deficiency

**DOI:** 10.1186/s13023-016-0522-z

**Published:** 2017-01-18

**Authors:** Tessa Wassenberg, Marta Molero-Luis, Kathrin Jeltsch, Georg F. Hoffmann, Birgit Assmann, Nenad Blau, Angeles Garcia-Cazorla, Rafael Artuch, Roser Pons, Toni S. Pearson, Vincenco Leuzzi, Mario Mastrangelo, Phillip L. Pearl, Wang Tso Lee, Manju A. Kurian, Simon Heales, Lisa Flint, Marcel Verbeek, Michèl Willemsen, Thomas Opladen

**Affiliations:** 1 Department of Neurology and Child Neurology, Radboud university medical center, Donders Institute for Brain, Cognition and Behaviour, Nijmegen, The Netherlands; 2 Department of Clinical Biochemistry, CIBERER-ISCIII, Hospital Sant Joan de Déu Barcelona, Barcelona, Spain; 3 Department of Child Neurology and Metabolic Disorders, University Children’s Hospital, Heidelberg, Germany; 4 Dietmar-Hopp Metabolic Center, University Children’s Hospital Heidelberg, Heidelberg, Germany; 5 Department of Child Neurology, CIBERER-ISCIII, Hospital Sant Joan de Déu Barcelona, Barcelona, Spain; 6 First Department of Pediatrics, Pediatric Neurology Unit, Agia Sofia Hospital, National and Kapodistrian University of Athens, Athens, Greece; 7 Department of Neurology, Washington University School of Medicine, St. Louis, USA; 8 Department of Pediatrics and Child Neuropsychiatry, Sapienza Università di Roma, Rome, Italy; 9 Department of Epilepsy and Clinical Neurophysiology, Boston Children’s Hospital, Harvard Medical School, Boston, USA; 10 Department of Pediatrics, National Taiwan University Hospital, Taipei, Taiwan; 11 Developmental Neurosciences, UCL- Institute of Child Health and Department of Neurology, Great Ormond Street Hospital for Children NHS Foundations Trust, London, UK; 12 Laboratory Medicine, Great Ormond Street Hospital and Neurometabolic Unit, National Hospital, London, UK; 13 AADC research trust, London, UK; 14 Department Laboratory Medicine, Alzheimer Centre, Radboud university medical center, Nijmegen, The Netherlands

**Keywords:** Aromatic l-amino acid decarboxylase deficiency, AADC deficiency, Neurotransmitter, Dopamine, Serotonin, Guideline, Infantile dystonia-parkinsonism, SIGN, GRADE

## Abstract

Aromatic L-amino acid decarboxylase deficiency (AADCD) is a rare, autosomal recessive neurometabolic disorder that leads to a severe combined deficiency of serotonin, dopamine, norepinephrine and epinephrine. Onset is early in life, and key clinical symptoms are hypotonia, movement disorders (oculogyric crisis, dystonia, and hypokinesia), developmental delay, and autonomic symptoms.

In this consensus guideline, representatives of the International Working Group on Neurotransmitter Related Disorders (iNTD) and patient representatives evaluated all available evidence for diagnosis and treatment of AADCD and made recommendations using SIGN and GRADE methodology. In the face of limited definitive evidence, we constructed practical recommendations on clinical diagnosis, laboratory diagnosis, imaging and electroencephalograpy, medical treatments and non-medical treatments. Furthermore, we identified topics for further research. We believe this guideline will improve the care for AADCD patients around the world whilst promoting general awareness of this rare disease.

**Electronic supplementary material:**

The online version of this article (doi:10.1186/s13023-016-0522-z) contains supplementary material, which is available to authorized users.

## German abstract

Der Aromatische L-Aminosäuren Decarboxylase Mangel (AADCD) ist eine seltene autosomal rezessive neurometabolische Störung, die zu einem schweren kombinierten Mangel an Serotonin, Dopamin, Norepinephrin und Epinephrin führt. Die Symptome setzen in einer frühen Phase des Lebens ein. Die klinischen Hauptsymptome sind muskuläre Hypotonie, Bewegungsstörungen (Okkulogyre Krisen, Dystonie und Hypokinesie), Entwicklungsverzögerung und autonome Symptome.

In diesen Konsensus basierten Leitlinien haben Mitglieder der “International Working Group on Neurotransmitter Related Disorders (iNTD)” sowie Patientenvertreter die verfügbare Evidenz für die Diagnose und Behandlung von AADCD ausgewertet und evidenzbasierte Empfehlungen nach den Methoden von SIGN und GRADE formuliert. Stets im Bewusstsein der limitierten Evidenz haben wir praktische Empfehlungen für die klinische Diagnose, Labordiagnose, Bildgebung und EEG, medizinische und nicht medizinische Behandlung formuliert. Des Weiteren haben wir Themen identifiziert, die der weiteren Forschung bedürfen. Wir glauben, dass diese Leitlinie die Behandlung von Patienten mit AADCD verbessert und gleichzeitig das Bewusstsein für diese seltene Erkrankung geschärft wird.

## Spanish abstract

La deficiencia de la descarboxilasa de aminoácidos aromáticos (AADCD) es una enfermedad neurometabólica minoritaria de herencia autosómica recesiva que causa un defecto grave de dopamina, serotonina, epinefrina y norepinefrina. El inicio de la enfermedad es precoz, y sus síntomas principales incluyen hipotonía, trastornos del movimiento (crisis oculógiras, distonía e hipocinesia), retraso del desarrollo y signos disautonómicos.

En esta guía de consenso han participado representantes del grupo de trabajo internacional sobre trastornos de la neurotransmisión (iNTD) y representantes de las asociaciones de pacientes, que han evaluado todas las evidencias existentes en relación al diagnóstico y tratamiento de la AADCD. Las recomnedaciones se han realizado siguiendo la metodología SIGN y GRADE. A pesar de los bajos niveles de evidencia existentes, se han podido establecer recomendaciones prácticas y que pueden ser muy útiles sobre el diagnóstico clínico y de laboratorio, las pruebas de neuroimagen y electroencefalográficas y sobre tratamientos tanto médicos como de soporte. Se han identificado además temas que pueden ser interesantes a desarrollar en el campo de la investigación de la AADCD.

En conclusión, pensamos que esta guía aumentará la calidad en los cuidados de los pacientes con AADCD en todo el mundo, y que aumentará el conocimiento de esta rara enfermedad.

## Background

**Fig. 1:**
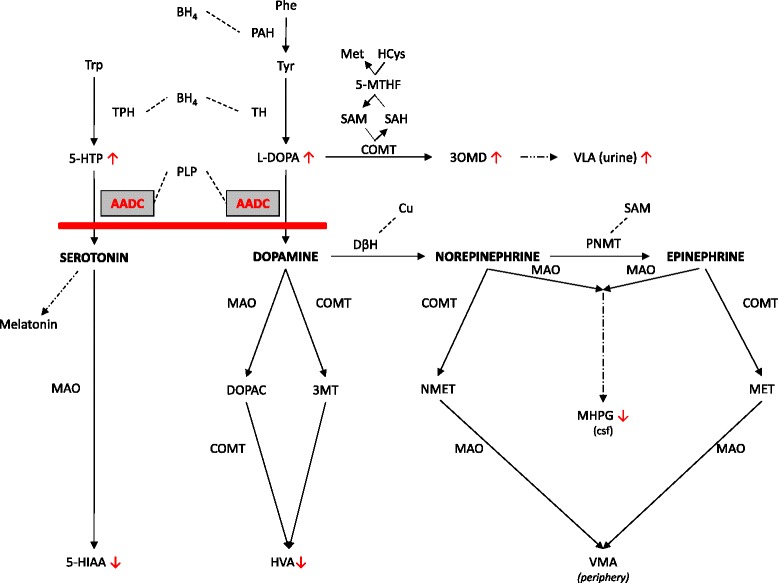
Biosynthesis and breakdown of serotonin and the catecholamines, and the metabolic block in AADC deficiency. Simplified scheme of the biosynthesis and breakdown of serotonin and the catecholamines (dopamine, norepinephrine and epinephrine), and melatonin synthesis. Cofactors (BH_4_, PLP, Cu) and methyldonor (SAM) are connected to the respective enzyme with dashed lines. Dashed arrows do not show intermediate steps. The metabolic block caused by AADC deficiency is shown as a red bar. Metabolites above the block are increased, metabolites below the block are decreased, indicated by red arrows. The implication of 5-MTHF in L-dopa to 3-OMD metabolism is shown in a simplified manner. Norepinephrine and epinephrine are broken down to NMET and MET only in the periphery. In CSF, the main metabolite of norepinephrine and epinephrine is MHPG. Abbreviations: AADC: aromatic l-amino acid decarboxylase; BH_4_: tetrahydrobiopterin; COMT: catechol O-methyl transferase; CSF: cerebrospinal fluid; Cu: cupper; DβH: dopamine beta hydroxylase; DOPAC: dihydroxyphenylacetic acid; HCys: homocysteine; 5-HIAA: 5-hydroxyindoleacetic acid; 5-HTP: 5-hydroxytryptophan; HVA: homovanillic acid; L-Dopa: 3,4-dihydroxyphenylalanine; MAO: monoamine oxidase; MET: metanephrine; Met: metionine; MHPG: 3-methoxy 4-hydroxyphenylglycol; 3MT: 3-Metyramine; 5-MTHF: methyltetrahydrofolate; NMET: normetanephrine; 3-OMD: 3-O-methyldopa (=3-methoxytyrosine); Phe: phenylalanine; PhH: phenylalanine hydroxylase; PNMT: phenylethanolamine N-methyltransferase; SAH: S-adenosylhomocysteine; SAM: s-adenosylmethionine; TH: tyrosine hydroxylase; TrH: tryptophan hydroxylase; Tryp: tryptophan; Tyr: tyrosine; VLA: vanillactic acid; VMA: vanillmandelic acid: Vit B6 vitamin B6 (pyridoxine)

Aromatic L-amino acid decarboxylase (AADC; EC 4.1.1.28) is the final enzyme in the biosynthesis of the monoamine neurotransmitters serotonin and dopamine, and dopamine is the precursor for norepinephrine and epinephrine. AADC deficiency (AADCD; OMIM 608643) is a rare, autosomal recessive neurometabolic disorder that leads to a severe combined deficiency of serotonin, dopamine, norepinephrine and epinephrine (Fig. 1) [1]. About 100 patients have been described in case reports or case series since the initial description of the index family in 1990 [2, 3]. The global incidence of AADCD is unknown, and there are no newborn screening programs, but it is more prevalent in certain Asian (especially Taiwanese and Japanese) populations, probably due to a founder effect [4]. Symptom onset typically occurs during the first months of life [2]. While most patients present a severe phenotype with early onset hypotonia, oculogyric crises, ptosis, dystonia, hypokinesia, impaired development and autonomic dysfunction [2], a few patients with a milder disease course are known [5].

Due to the rarity of AADCD, there is limited international clinical expertise for evidence-based management. Treatment response is often disappointing and treatment strategies vary between single expert centers [6]. Some patients are treated with a variety of different drugs, while others only receive one or two different drug classes [2]. A small subset of patients shows a good response to L-Dopa [7] or dopamine agonists [5], but often patients show no or poor response [2]. For most patients, treatment response cannot be predicted.

To improve care for patients with neurotransmitter related disorders, including AADCD, the International Working Group on Neurotransmitter Related Disorders (iNTD) was founded in 2013 [8]. Today, iNTD is a growing worldwide network of 38 neurometabolic centers from 24 countries. In addition to keeping a patient registry [9], one of its goals is to develop consensus care guidelines for neurotransmitter related disorders by pooling all published evidence and experience of leading expert centers. This guideline on diagnosis and treatment of AADCD is the first guideline developed by iNTD.

In this guideline, developed with the Scottish Intercollegiate Guideline Network (SIGN) methodology, we present the key clinical symptoms and the recommended strategy for diagnosis and management of AADCD based on a systematic review of the literature and consensus meetings of the iNTD guideline working group. The guideline is intended for metabolic specialists, child and adult neurologists, pediatricians, intensive care specialists, nurses, and paramedical specialists involved in the care of patients with AADCD.

## Methods

### Composition of the guideline working group and timeline

An executive committee (TO (chairman), TW (secretary), MM and KJ) was appointed to oversee the guideline development process and to function as coordinators for the subgroups focusing on different topics. The guideline working group consisted of 13 child neurologists (TW, GH, BA, AC, MK, WL, RP, TP, VL, MMa, PP, MW, TO), 5 biochemists (MM, NB, RA, SH, MV), and 1 research project manager (KJ) from several European countries, the USA and Taiwan. All group members are affiliated to iNTD and are experienced in the diagnosis and treatment of AADCD. Furthermore, a representative of the European AADCD patient organization (LF, AADC Research Trust) participated in guideline development. Finally, according to SIGN guidelines, two external academic reviewers with expertise in neurometabolic and movement disorders (Ron Wevers, Nijmegen, the Netherlands and Russell Dale, Sydney, Australia), and one additional lay reviewer were asked to comment on the draft before submission. The final version of the guideline was pilot-tested for use in clinical practice by non-specialist neurologists in training. A preliminary guideline working group meeting took place in November 2014 (London, UK) during the AADC Research Trust Conference. The start-up meeting took place in Barcelona, Spain (February 2015). Further face-to-face meetings were held in Heidelberg, Germany (September 2015, executive committee only), in Venice, Italy (November 2015), and a final wrap-up meeting was held during the AADC Research Trust Conference in May 2016 (London, UK).

### Developing topics and key questions

During the start-up meeting, the initial list of key questions compiled by TW and TO was discussed and refined. The group was divided in subgroups focusing on the following topics: (I) Clinical Presentation; (IIa) Diagnosis: laboratory tests; (IIb) Diagnosis: imaging; (III) Treatment; (IV) Complications and Long-Term Management; (V) Social Issues and Transition; and (VI) Special Situations. See Additional file 1: Table S1 for the final list of key questions.

### Systematic literature search

A systematic literature review on AADCD was performed through December 2015 on Pubmed, Cochrane database, and the Cinahl database, using “aromatic (l) amino acid decarboxylase deficiency” and “AADC deficiency” as search terms. No language or data filters were used. The literature database on the official website of the AADC research trust was searched for applicable articles [10]. The reference list of the large case series of Brun [2] was screened for additional sources. Furthermore, reviews on monoamine neurotransmitter deficiencies were searched using Pubmed, search term “monoamine neurotransmitter disorders’, limits: review, English, from 1990. The WHO registry for clinical trials [11] and the NIH registry for clinical trials [12] were searched. Finally, reference lists from review articles and key case series were screened for additional hits and members of the guideline group were asked to suggest relevant book chapters. Because of the limited number of publications on AADCD, conference abstracts from posters or symposia were included for literature review, but only if they were not followed by a peer reviewed publication from the group. The flow chart with results of the literature search can be found as Additional file 2: Figure S1.

### Grading of evidence and recommendations

The guideline was developed according to SIGN [13]. For rating the quality of evidence and defining strength of recommendations, SIGN is committed to using the GRADE methodology [14–17]. Level of evidence of individual studies was rated in a range from 4 (lowest) to 1++ (highest) (Table 1). Specific outcomes (*e.g*. effect of a specific drug on hypotonia) were described with a certain level of evidence (very low, low, moderate or high) for the total body of evidence. Recommendations were rated as strong (for or against), conditional (for or against), or recommendation for further research (Table 2). Furthermore, Good Practice Points (GPP) were formulated based on the clinical experience of the guideline development group. Relevant papers were evaluated by at least two guideline working group members. Before and during meetings, the guideline group members were trained on standardized literature evaluation using SIGN/ GRADE methodologies. All recommendations were discussed for consensus during guideline group meetings.

**Table 1:** Levels of evidence according to SIGN

Levels of evidence	
1++	High quality meta-analyses, systematic reviews of RCTs or RCTs with a very low risk of bias
1+	Well conducted meta-analyses, systematic reviews, or RCTs with a low risk of bias
1-	Meta-analyses, systematic reviews, or RCTs with a high risk of bias
2++	High quality systematic reviews of case control or cohort studies
	High quality case control or cohort studies with a very low risk of confounding or bias and a high probability that the relationship is causal
2+	Well conducted case control or cohort studies with a low risk of confounding bias and a moderate probability that the relationship is causal
2-	Case control or cohort studies with a high risk of confounding or bias and a significant risk that the relationship is not causal
3	Non-analytic studies, e.g. case reports, case series
4	Expert opinion

**Table 2:** Forms of recommendations according to GRADE

Judgment	Recommendation
Undesirable consequences clearly outweigh desirable consequences	Strong recommendation against
Undesirable consequences probably outweigh desirable consequences	Conditional recommendation against
Balance between desirable and undesirable consequences is closely balanced or uncertain	Recommendation for research and possibly conditional recommendation for use restricted to trials
Desirable consequences probably outweigh undesirable consequences	Conditional recommendation for
Desirable consequences clearly outweigh undesirable consequences	Strong recommendation for

### Disclaimer

The purpose of this guideline is to improve care for patients with AADCD and is based on the best available evidence. However, AADCD being a very rare disorder, the body of evidence encompasses mainly non-analytical studies and case reports. Therefore, many recommendations reflect expert opinion. This guideline is meant to give a solid foundation to caregivers who look after AADCD patients, but should never replace sensible, well-informed clinical care.

## Part I: Clinical presentation

### Number of reports; literature search

In addition to the 78 patients reported in the large case series of Brun et al in 2010 [2], 39 individual patients were identified in a total of 13 reports [3, 5, 18–28]. A further 22 cases, partly described in conference abstracts, could not with certainty be distinguished as individual patients [24, 29–33]. In other words, 117 to 139 AADCD patients have been described so far, and 117 patients were used for calculations of patient characteristics. The comprehensiveness of the clinical information and the amount of biochemical and molecular data available differed substantially between included reports. Six families with multiple affected members were described [1, 7, 34–37]. Of the 117 patients clearly identified as individual AADCD cases, 56 were male, 42 were female, and in 19 gender was not reported.

### Ethnic origin

Many reported patients (50/117) are Asian. It is known that there is an increased prevalence of AADCD in patients from southern Chinese descent, especially from Taiwan and Japan, due to the founder variant IVS6 + 4A > T [4]. Other reported ethnic origins are: Caucasian (*n* = 33), Arabic (*n* = 4), Iranian (*n* = 1) and Jewish (*n* = 2). Ethnic origin was not described or remained uncertain in 21 cases.

**R#1 (strong):** There should be an increased level of awareness for AADCD in patients of Chinese/Taiwanese/Japanese descent, because of the presence of a founder variant (IVS6 + 4A > T).

### Age of onset and age of diagnosis

The age of onset of signs and symptoms, clearly documented in 68 cases only, was within the first year of life (mean: 2.7 months, SD ± 2.7). Eleven patients presented in the neonatal period with hypotonia, or floppiness. Based on available data (mostly level 3, with non-standardized methods of reporting symptoms), it was not possible to describe the precise age of onset and duration of every symptom associated with AADCD. Table 3 summarizes the best available data from published cases regarding the onset of symptoms. Despite the early onset of symptoms, mean age of diagnosis was 3.5 years (median 13 months, range 2 months – 23 years, 26 % missing data).

**Table 3:** Symptoms and signs described in AADC deficiency

		Symptom/ sign	Neo natal	Infancy	Child hood	Adolescence	Adulthood
CNS	Tone regulation	Floppy infant	+	++			
		Hypotonia (mainly truncal)	+	++	++	++	++
		Poor head control	+	+	+	+	+
		Hypertonia (mainly limbs)	+	+	+	+	+
	Movement disorders	Dyskinesia (eg hyperkinesia, chorea, athetosis)	±	+	+	+	+
		Dystonia	-	++	++	++	++
		Oculogyric crisis	±	++	++	++	++
		Hypokinesia and/ or bradykinesia	±	++	++	++	++
		Myoclonus	±	±	±	±	±
		Tremor	±	±	±	±	±
	Developmental Delay	Delayed motor development	±	++	++	++	++
		Delayed cognitive development		±	+	+	+
		Delayed speech development		±	+	+	+
	Behavioural Problems	Irritability	++	++	+	+	+
		Autistic features			±	±	±
		Dysphoria/ Mood problems	±	±	±	±	±
		Excessive crying	+	++	+	-	-
	Sleeping Problems	Insomnia and or hypersomnia		+	+	+	+
	Other	Epileptic seizures	-	±	±	±	±
		Fatiguability	±	±	±	±	±
		Diurnal fluctuation		±	±	±	±
		Dysarthria			±	±	±
		Poor eye fixation		+	+	+	+
		Increased startle	±	±	±	±	±
ANS	Eyes	Ptosis	+	+	+	+	+
		Miosis	±	±	±	±	±
	Upper respiratory tract	Nasal congestion	-	+	+	±	±
		Excessive drooling	-	+	+	±	±
		Stridor	±	±	±	±	±
	Skin	Excessive sweating	-	+	+	+	+
	Homeostasis	Temperature instability	+	+	+	+	+
	Cardiovascular	(Orthostatic) Hypotension	-	-	±	+	+
		Bradycardia	±	±	±	±	±
		Heart rhytm abnormalities	±	±	±	±	
	Gastrointestinal	Diarrhea	±	+	+	+	±
		Obstipation	±	+	+	+	±
Metabolic/ endocrine		Hypoglycemia	±	±	±	-	-
		Hyperprolactinemia	±	±	±	±	±
General		Feeding/ Swallowing Problems	+	+	+	+	+
		Gastrointestinal reflux	+	+	+	±	±
		Gastrointestinal problems unspecified	+	++	+	+	+
		Failure to thrive	±	+	+	+	
		Contractures	-	-	-	±	±
		Small hand and feet	±	±	±	±	±

Best available evidence for all published clinical symptoms and the age of occurrence (additional literature used: [77]). *CNS* Central Nervous System. *ANS* autonomic nervous system. ++ very often (key symptom); + often; ± sometimes; - not expected

### Key symptoms and signs

Key symptoms of AADCD are: hypotonia (present in *n* = 91 patients, absent in *n* = 3 patients, not mentioned in *n* = 23 patients), movement disorders (present *n* = 99, absent *n* = 3, not mentioned *n* = 15), developmental delay (present *n* = 84, absent in *n* = 1 only [18], not mentioned *n* = 32), and autonomic symptoms (present *n* = 76, absent *n* = 3, not mentioned *n* = 38). All key symptoms ranged in severity from mild to very severe. Hypotonia is most often axial, and sometimes accompanied by limb hypertonia. Movement disorders most often described are oculogyric crises (*n* = 91), dystonia (*n* = 63), and hypokinesia (*n* = 40). Most prominent autonomic signs are ptosis, excessive sweating, and nasal congestion. Hypotension or orthostatic hypotension was reported in 15 patients, with onset mostly in late childhood or adolescence. In 14 patients, blood pressure was reported to be normal. A complete list of movement disorders, autonomic symptoms and signs, and other clinical characteristics is given in Table 3.

**R#2(strong):** In children with unexplained hypotonia, movement disorders (especially oculogyric crisis), developmental delay, and autonomic symptoms, AADCD should be considered.

### Other neurological findings

Epileptic seizures were described in 9 patients [2, 7, 22, 38–40]. Behavioral problems can be a great burden to patients and caregivers but are not well defined in the literature. They were reported to be present in 41 cases, mostly described as irritability, excessive crying, dysphoria [2, 40, 41] and autistic features [31]. Sleep disturbance (both insomnia and hypersomnia) was described in 34 patients. Some patients may suffer from severe sleep apnea, which may be lethal (personal observation WL). In some patients, diurnal fluctuations and improvement after sleep, a classical finding for many neurotransmitter disorders [6], is reported. On neurological examination, deep tendon reflexes can be decreased, normal or increased. Pathological reflexes including Babinski sign are sometimes reported.

**R#3 (research):** Behavioral symptoms and sleep disturbances in AADCD should be better defined, to improve patient care.

### Additional clinical findings

Gastrointestinal problems can be prominent in AADCD. Symptoms can include diarrhea, constipation, gastroesophageal reflux, and feeding difficulties. Many patients receive and benefit from a gastrostomy (expert experience). Failure to thrive and short stature is often reported. In 13 patients, intermittent hypoglycemia was reported, at birth or in the first five years of life. Hypoglycemia can occur during intercurrent illnesses [41]. In one patient, hypoglycemia with reduced level of consciousness was found after sedation for MRI [42]. There are no reports of growth hormone deficiency, thyroid deficiency, or other hormone deficiencies in AADCD.

### Phenotypic spectrum and clinical course

Based on clinical description, cases were broadly classified as mild (mild delay in developmental milestones, ambulatory without assistance, mild intellectual disability), severe (no or very limited developmental milestones, fully dependent), and moderate (in between). Six cases could be classified as mild (reported in [5, 18, 19, 35, 43]), 15 as moderate (reported in [2, 5, 7, 20, 22, 31, 35, 40, 44, 45]), and 82 as severe. For 14 cases, insufficient information about the clinical picture was available. Importantly, a mild phenotype can present predominantly with autonomic symptoms (diarrhea, episodic hypoglycemia, nasal congestion) and without evident movement disorders [18]. Two adult sisters had a very atypical clinical course, with hypotonia at birth, moderate to severe motor developmental delay in infancy, and mild to moderate symptoms in childhood, with improvement during puberty [5]. The two other patients who were diagnosed as adults did not show transient improvement in puberty, but rather a deteriorating clinical picture [7]. Deterioration with loss of skills, e.g. regression of language skills [20, 22], is described in some patients but in the majority of cases, there is no evident progressive clinical course with loss of function described. A decline in motor function can sometimes be due to secondary factors like joint contractures [46].

**R#4 (conditional):** The phenotypic spectrum of AADCD is broad and can range from very severe to relatively mild. AADCD should also be considered in patients with autonomic symptoms without obvious movement disorders.

**R#5 (strong):** A deteriorating clinical course is not expected in patients with AADCD and should trigger a diagnostic work-up for other diseases.

### Phenotype correlations with genotype or biochemical phenotype

There are >50 different *DDC* gene disease causing variants described in AADCD but clear genotype/ phenotype correlations could not be established. However, patients with the founder splice variant IVS6 + 4A > T (36 patients in total, 26 with homozygous variants) all had a severe phenotype without reaching clear developmental milestones, except for two sisters with the compound heterozygous variants p.[R285W];[IVS6 + 4A > T] and a mild to moderate clinical picture with response to treatment [35]. The variant p.[R285W],c.[853C > T] is not reported in other patients. Gender is not associated with phenotype (severe phenotype in 72 % of females and 77 % of males).

There is evidence for a genotype/ treatment response correlation in two families with different L-Dopa binding site variants and a convincing response to L-Dopa. Three siblings, extensively represented in the literature, with a homozygous p.[G102S], c.[304G > A] variant became able to walk independently (*n* = 1) or with assistance (*n* = 2) and showed improvement of dystonia, truncal hypotonia and speech after L-Dopa treatment was started [7, 40, 47]. More recently, a patient with other variants affecting the L-Dopa binding site p.[R347Q];[R160W], c.[1040G > A];[478C > T], also showed a good response to L-Dopa [19]. Currently, therapeutic implications of other *DDC* gene variants in AADCD are being investigated [48].

There is overlap in cerebrospinal fluid (CSF) values of biogenic amines in mild, moderate and severe cases without a clear correlation of the biochemical (CSF) and clinical phenotype. Plasma AADC enzyme activity does not correlate with clinical phenotype; in both mild and severe cases it can be below the detection limit of the assay.

**R#6 (conditional):** There are no clear genotype/ biochemical or clinical phenotype correlations in AADCD except for the homozygous IVS6 + 4A > T splice variant that is associated with a severe phenotype in all cases reported to date, and rare L-Dopa binding site variants that are associated with L-Dopa responsiveness.

## Part IIa: Diagnosis: laboratory tests

### Key diagnostic tests: CSF, AADC activity and genetic testing

#### Lumbar puncture

The typical CSF pattern in AADCD consists of (1) low levels of 5-hydroxyindoleacetic acid (5-HIAA), homovanillic acid (HVA) and 3-methoxy-4-hydroxyphenylglycol (MHPG), (2) normal pterins including neopterin and biopterin, and (3) high concentrations of 3-O-methyldopa (3-OMD), L-Dopa and 5-OH tryptophan (5-HTP). This reflects the metabolic block at the level of AADC (Fig. 1). Low HVA and 5-HIAA was reported in 99 % of patients. Only two sisters with a mild phenotype had normal HVA (124 and 169 nmol/L; ref 98-450 nmol/L), one of them also had normal 5-HIAA (50 nmol/L; ref 45-135 nmol/L). In both patients, 3-OMD and -5-HTP were increased and MHPG was decreased [5]. In only one patient, normal MHPG was reported, with decreased HVA and 5-HIAA [19]. Normal CSF pterins (neopterin, dihydrobiopterin and tetrahydrobiopterin) are essential to differentiate AADCD from the tetrahydrobiopterin disorders [6].

The CSF profile of AADCD may be similar to the profile found in pyridox(am)ine 5-phosphate (PNPO) deficiency, in which there is a secondary failure of AADC due to a deficiency of its cofactor pyridoxal phosphate (PLP). However, additional findings in this disorder are very low PLP, and increased glycine and threonine in CSF. Furthermore, the clinical picture of PNPO deficiency, namely a severe neonatal epileptic encephalopathy, is different from the clinical presentation of AADCD [49, 50].

Mildly decreased CSF 5-methyltetrahydrofolate (5-MTHF) was reported in only 1 patient with AADCD [47], and 5-MTHF levels decreased during L-Dopa treatment in 3 patients [40]. It was proposed that this could be due to a depletion of CSF s-adenosylmethionine (SAM) in states with increased L-Dopa concentrations [51], but serial CSF measurements for 5-MTHF have not been performed in other patients.

Neurotransmitter metabolite analysis is performed in a limited number of specialized laboratories. For an online list of iNTD affiliated laboratories, see [8]. Collection and handling of CSF should be performed strictly following standardized procedures to ensure correct interpretation of results. For a review see Hyland [52].

**R#7 (strong):** AADCD can be diagnosed in CSF. Specific measurements in CSF should include the core metabolites HVA, 5-HIAA, 3-OMD, L-Dopa, and 5-HTP, and pterins. If available, 5-MTHF and PLP should also be measured. Reduced 5-HIAA and HVA, and elevated 3-OMD, L-Dopa and 5-HTP, with normal pterins, point to AADCD.

**R#8 (GPP):** CSF measurements should always include standard measurements (cells, protein, glucose, lactate). Collection and handling of CSF should be performed strictly following standardized procedures to ensure correct interpretation of results.

#### AADC activity in plasma

Enzyme assays measuring AADC activity in plasma can be performed using both L-Dopa and 5-HTP as substrate. Since L-Dopa gives a higher analytical yield it is used as the standard method. All 57 papers that reported results of plasma AADC activity assays, described decreased activity in patients with AADCD. In about 30 % of reported cases, AADC activity was below detection limits. The highest reported residual enzyme activity in AADCD was 12 pmol/mL/min (33 % of the lower limit of normal), reported in 1 patient [41]. AADC activity is also decreased in heterozygous carriers (35-40 % of normal) [18, 34, 35, 53]. For an online list of iNTD affiliated laboratories that perform AADC activity assays, see [8]

**R#9 (strong):** AADCD can be diagnosed by demonstrating severely decreased AADC activity in plasma. Plasma AADC activity is moderately reduced in heterozygous carriers.

#### Molecular diagnosis

About 50 disease causing *DDC* variants have been described so far: 39 substitution variants, 2 nonsense, 5 deletions, 1 insertion and 4 splice variants. For an up to date list of *DDC* variants, see online locus-specific database on PNDdb [54]. In only two AADCD patients with the classical CSF profile and decreased AADC activity in plasma, no variants of the *DDC* gene were found despite Sanger analysis of the coding region, the exon/intron boundaries and flanking untranslated regions [39, 41]. In two other patients (siblings),with the classical CSF profiles and very low plasma AADC enzyme activity, only one variant was detected [36]. It is assumed that in these cases a second variant may be intronic, within the promoter region or a heterozygous deletion or duplication, undetectable by current standard diagnostic techniques. Two recent reports detected AADCD patients using whole exome sequencing [20, 55].

**R#10 (strong):** In the great majority of patients, AADCD can be genetically confirmed.

### Concluding statements regarding key diagnostic tests

**R#11 (strong):** There are three core diagnostic keys for identifying AADCD:

1. low CSF levels of 5-HIAA, HVA, and MHPG, increased CSF levels of 3-OMD, L-Dopa and 5-HTP, and normal CSF pterins
2. compound heterozygous or homozygous pathogenic variants in the *DDC* gene
3. decreased AADC enzyme activity in plasma

To diagnose AADCD, genetic testing should be performed and at least two out of three core diagnostic tests should be positive.

**R#12 (strong):** If genetic diagnosis is performed as the first step (e.g. whole exome sequencing or affected family member), functional confirmation should be completed by measuring AADC enzyme activity in plasma and/or neurotransmitter metabolites in CSF.

**R#13 (conditional):** If local resources allow, we recommend performing all three key diagnostic tests in patients with this rare disorder.

**R#14 (GPP):** The results of CSF analysis and plasma AADC activity measurement are generally available before results of genetic testing. Genetic confirmation should not be awaited before initiating therapy.

### Other diagnostic tests in AADCD

#### Prolactin

Prolactin in blood can be increased in dopamine biosynthesis disorders, because dopamine serves as an inhibitor of prolactin secretion [6]. Prolactin was reported in 13 AADCD patients. Elevated prolactin was found in 6 patients; in the others it was normal. Also, members of the guideline group reported normal prolactin in multiple AADCD patients (non-published observations).

**R#15 (research):** Normal prolactin level in blood does not exclude AADCD. To better understand this mechanism we recommend further research on this topic.

#### Neurotransmitter (metabolites) in blood

Whole blood serotonin was reported in 15 patients and found to be decreased in all of them [1, 34, 39, 40, 56]. Plasma monoamine and monoamine metabolites (L-Dopa, 3-OMD, vanillactic acid (VLA), HVA, vanillylmandelic acid (VMA), MHPG, norepinephrine, epinephrine, and 5-HTP) are reported in a very limited number of patients and it is not possible to draw conclusions about the diagnostic accuracy of these tests. Plasma measurements cannot replace measurements in CSF.

**R#16 (strong):** Measurement of catecholamine metabolites in blood are not useful in routine clinical practice, because it neither diagnoses nor excludes AADCD.

#### Dried blood spot measurement of 3-OMD

One report studied the use of dried blood spot (DBS) testing of 3-OMD in 15 patients [57]. All samples showed an at least 15-fold increase of 3-OMD. In 6 patients, newborn values were available, showing very high levels of 3-OMD. However, 3-OMD is also increased in PNPO deficiency. There are no other reports on this subject available. If (newborn) screening can reliably diagnose AADCD, this will greatly improve the diagnostic delay that is often encountered now. A clinical trial on newborn screening of AADCD is currently recruiting patients in Taiwan [12].

**R#17 (research):** The development, diagnostic accuracy, and cost effectiveness of DBS analysis of 3-OMD for (newborn) screening for AADCD should be further investigated.

#### Urine measurements of neurotransmitter metabolites

Increased urine vanillactic acid (VLA) levels, measured by organic acid analysis, are reported in AADCD. However, this elevation is often subtle and easily missed if not specifically looked for in a specialised laboratory [2].

Dopamine, HVA and vanilylmandelic acid (VMA) in urine are not reliable as biomarkers in AADCD because they can either be normal, decreased or increased [2, 26, 58]. This is due to very high AADC activity within the kidneys, accounting for sufficient residual AADC activity to form dopamine and its metabolites [27].

**R#18 (conditional):** If urinary VLA is increased, AADCD should be considered. However, normal levels do not exclude the diagnosis.

**R#19 (strong):** Measurement of catecholamine metabolites in urine is not useful as a diagnostic test in clinical practice, because it can neither diagnose nor exclude AADCD.

#### Other diagnostic tests

Melatonin is formed from serotonin and would be expected to be decreased in AADCD. Theoretically, it is therefore a possible diagnostic tool for this patient group, but no reports on melatonin measurements are available. One recent report used global metabolomics assisted pathway screening with plasma samples in one AADCD patient, and showed an increased 3-OMD. This finding was also seen in subjects using L-Dopa/ carbidopa medication [55].

**R#20 (research):** Other diagnostic tests that might increase knowledge on AADCD and/ or serve as possible biomarkers are melatonin and broad metabolomic screening for specific AADCD related markers.

## Part IIb: Diagnosis: imaging and electroencephalography

### Magnetic resonance imaging (MRI) of the brain

Brain imaging was performed in 38 cases (36 MRI, 2 CT), and was described as normal in 25. In the remaining cases, described abnormalities were heterogeneous, including: diffuse mild cerebral atrophy [2, 38], cortical atrophy [41], reduced prefrontal volume with normal myelination and normal basal ganglia [36], focal demyelination changes over bilateral frontal and left parietal area [4], leukomalacia [4], degenerative changes of white matter, thinning of corpus callosum, prominent ventricular bodies, leukodystrophy-like patterns, and hypomyelination [2]. Since there is no specific MRI pattern, imaging is not helpful in the diagnosis of AADCD. However, it is usually necessary in the work-up of a patient with neurodevelopmental delay to exclude other conditions in the differential diagnosis. Following standards of good clinical care, neuroimaging is always indicated if there is an unexpected deviation of clinical course in a diagnosed AADCD patient (see also R #5).

**R#21 (conditional):** Routine imaging of the brain is not needed to diagnose AADCD.

**R#22 (GPP):** In the work-up of patients with neurodevelopmental delay, and in unexpected deviation of clinical course in AADCD patients, brain imaging should be considered.

### Electroencephalography (EEG)

EEG, reported in 28 cases, was described as normal in 21. In the remaining cases described abnormalities included: epileptiform abnormalities in a patient with clinical seizures [38], epileptiform abnormalities (paroxysmal discharges, (multifocal) sharp waves, polyspikes) without evident clinical seizures in three patients [2, 56, 59], unspecific changes [41], and rapid activity or slowing [2]. EEG is not needed to diagnose AADCD. However, if there is a clinical suspicion of epilepsy, to differentiate oculogyric crises from epileptic events [7], or in the general work-up of neurodevelopmental delay, EEG can be performed following local standards of good clinical care.

**R#23 (conditional):** EEG is not needed to diagnose AADCD.

**R#24 (GPP):** EEG can be used in the work-up of AADCD if there is a clinical suspicion of epilepsy, to differentiate oculogyric crises from epileptic events, or in the general work-up of neurodevelopmental delay.

### Other imaging modalities

A brain fluorodeoxyglucose (^18^F-FDG) PET scan was performed in only 1 patient [36], and demonstrated reduced uptake in the prefrontal cortex and bilateral basal ganglia. Striatal uptake of fluorodopa (FDOPA) on PET/CT was reported to be very low at baseline, and increased after gene therapy in 3 patients [24]. There were no published reports of DAT scans in AADCD.

**R#25 (research):** Other imaging modalities (FDG PET, FDOPA PET, DAT-scan) have no role in routine clinical care but can be useful in research settings, e.g. the assessment of the efficacy of gene therapy.

## Part III: Treatment

### IIIa: Medical treatment

#### Available evidence

Based on the literature evaluation, considered judgments leading to recommendations for use in clinical practice could be constructed for dopamine agonists, monoamine oxidase (MAO) inhibitors, pyridoxal phosphate, pyridoxine, anticholinergic agents, folinic acid, L-Dopa with carbidopa, L-Dopa without carbidopa, 5-hydroxytryptophan, benzodiazepines, melatonin, and selective serotonin reuptake inhibitors (SSRIs). For catechol O-methyl transferase (COMT)-inhibitors there was insufficient literature available to come to a recommendation. The quality of the total body of evidence for specific outcomes was rated as low or very low. There are currently no active medical treatment trials in AADCD registered.

### First line treatment

#### Dopamine agonists

Dopamine agonists activate postsynaptic dopamine receptors directly. Ergot-derived dopamine agonists with strong serotonergic (5HT2b) agonist action (pergolide and cabergoline) are strongly associated with cardiac valvulopathy and other fibrous complications [60], and should not be used in AADCD. Ergot-derived dopamine agonists without 5HT2b agonist action (bromocriptine) have a lower risk, although incidentally, pulmonary, retroperitoneal and (peri)cardial fibrosis have been described, with a dose-effect relation [61]. Non-ergot derived dopamine agonists are pramipexole, ropinirole, rotigotine (transdermal patches) and apomorphine (subcutaneous). The risk of fibrotic complications with non-ergot derived dopamine agonists is probably very low [62].

Use of dopamine agonists in AADCD was described in 58 individual cases, with bromocriptine being used most often (26 patients), followed by pergolide (11 patients). Reports on non-ergot derived dopamine agonists were fewer (4 patients on pramipexole, 4 on rotigotine, and 2 on ropinirole). One patient on cabergoline was described. In 9 patients the dopamine agonist was not specified. There are no reports of the use of subcutaneous apomorphine in AADCD. Positive responses (e.g. improvement of head control, hypotonia, oculogyric crises, voluntary movements and autonomic symptoms) have been reported for bromocriptine, pramipexole, rotigotine patches and pergolide. Also, neutral effects were reported. Reported side effects included irritability, weight loss, worsening of failure to thrive, vomiting, and also mild to severe dyskinesia [45]. Overall, benefits outweighed side effects in most cases. It is important to consider that many patients were treated simultaneously with more than one drug class, therefore assessment of the impact of a single drug is often problematic. For dose recommendations of dopamine agonists, see Table 4.

**R#26 (strong):** Dopamine agonists should be tried in the treatment of AADCD. Non-ergot derived dopamine agonists (pramipexole, ropinirole, rotigotine) are preferred.

**R#27 (strong):** Cabergoline and pergolide should not be used for the treatment of AADCD because of the high risk of fibrotic complications.

**R#28 (GPP):** Cardiac screening (see R#48) before and during treatment with bromocriptine (ergot derived dopamine-agonists) is indicated, because of the potential risk of cardiac fibrosis.

#### MAO inhibitors

MAO inhibitors prevent breakdown of dopamine and serotonin, thereby increasing monoamine availability. The effect of MAO inhibitors in AADCD was described for 31 cases in 17 studies. All patients had co-treatment with dopamine agonists and/ or pyridoxine. MAO inhibitors used were tranylcypromine (*n* = 14), selegiline (*n* = 7), phenelzine (*n* = 1), or unspecified (*n* = 9). No studies were found on rasagiline. The majority of studies described an improvement in at least one clinical endpoint (e.g. hypotonia), with no effects on others. Some studies reported no clinical improvement at all, e.g. Anselm et al [39], or only temporary improvement [40]. Side effects were rarely reported, apart from one patient who developed a dystonic crisis on withdrawal of tranylcypromine [45], and one patient with increased oculogyric crises [40]. For dose recommendations, see Table 4.

**R#29 (strong):** From the biochemical viewpoint there is strong recommendation for giving patients with AADCD a trial of MAO inhibitors, although there is little evidence of clinical benefit.

#### Pyridoxine / pyridoxal phosphate (PLP)

Pyridoxal phosphate (PLP), the active form of pyridoxine, is a cofactor of AADC (Fig. 1). Therefore, treatment with one of several available forms of vitamin B6 might increase residual activity of the AADC enzyme. Pyridoxine is more readily available and cheaper than PLP, therefore it is the form used most often in AADCD. It has been reported in about 50 individual patients. The use of PLP was described in only one study [22]. Pyridoxine monotherapy was reported in only 8 patients, of which only one patient (with a mild phenotype) showed a clear favorable response [18]. In patients with concurrent medications, a favorable effect was sometimes seen. In 19 patients, no clear response was described at all. Reported side effects were gastrointestinal complaints, sleeping problems and extreme motor restlessness in patients with very high doses of pyridoxine and concurrent treatment with L-Dopa. Pyridoxine and PLP can cause reversible polyneuropathy when used in high doses for long periods of time. For dose recommendations, see Table 4.

**R#30 (strong):** Vitamin B6 is considered a first line treatment from a biochemical standpoint, but dose limits should be respected because of possible side effects.

**R#31 (conditional):** Pyridoxine is preferred over PLP because of availability and costs. If it is not tolerated, PLP can be tried instead.

### Additional symptomatic treatment

#### Anticholinergic drugs

Anticholinergic drugs (e.g. trihexyphenidyl, benztropine, biperiden) are commonly used to treat certain movement disorders, especially parkinsonism [63] and dystonia [64]. Although their exact mechanism of action is not known, it is thought that they influence the relative imbalance between dopaminergic and cholinergic pathways. In AADCD, they can be used to treat autonomic symptoms, dystonia, and oculogyric crisis. The effect of anticholinergic medication is described in only 11 patients, who all received concurrent medications (dopamine agonist and/ or L-Dopa and/ or pyridoxine and/ or MAO-inhibitors). In the majority of cases there was an improvement of at least one clinical endpoint (e.g. hypotonia, excessive sweating, dystonia). Not all patients were reported to benefit, however. Side effects were reported in two articles, including significant sedation in one patient [39] and aggressive behavior in another [41]. For dose recommendations, see Table 4.

**R#32 (conditional):** Anticholinergic agents can be considered in AADCD, especially (additionally) to treat autonomic symptoms, dystonia, and oculogyric crisis.

#### Melatonin

There is very limited evidence for the use of melatonin in AADCD. It was reported in only 6 cases. An improvement in sleep pattern was described in 2 cases [41, 45], 1 patient showed no effect [21], and in 3 cases the clinical effect was not described [45]. No side effects have been reported although some members of the guideline working group reported experience with patients who had transient night terrors (unpublished observation). From a pathophysiological perspective, supplementation for disorders of sleep induction is reasonable because melatonin is formed from serotonin and therefore may be decreased in AADCD. Many AADCD patients suffer from sleeping disorders, and unpublished observations of the guideline group support the effect of melatonin in this patient group. For dose recommendations, see Table 4.

**R#33 (conditional):** Melatonin should be considered for the treatment of sleep disturbances in AADCD.

#### Benzodiazepines

There is very limited evidence for the use of benzodiazepines in AADCD, since it was reported in only 4 patients [38, 42, 65]. In one patient, there was a slight improvement of dystonia on clobazam [65]. In another patient, rectal diazepam was effective in the treatment of prolonged oculogyric crises [42]. General dose recommendations can be used.

**R#34 (conditional):** Benzodiazepines, especially when used intermittently, can be considered in specific settings, e.g. in sustained oculogyric or dystonic crises.

#### Other symptomatic treatment

Although no reports specifically evaluate the use of alpha-adrenoreceptor nose drops in AADCD, its value is evident in clinical practice to treat nasal congestion. To reduce side effects of long-term application, the lowest possible dose should be used. Application should be accompanied by local care, e.g. with dexpanthenol containing ointments. During long-term treatment an intermittent application of a topical steroid (e.g. fluticasone; one spray in each nostril per day for 6 weeks; maximal twice per year) can restore the nose drops effect and reduce the necessary dose (expert opinion). Clonidine, an imidazoline and alpha-2 agonist, can be used for irritability and sleep disturbance [6]. For dose recommendations, see Table 4. Botulinum toxin injections can be used in the treatment of dystonia. We have limited clinical experience with its use in AADCD.

### Other treatment options

#### L-Dopa with or without carbidopa

Patients with AADCD already have highly elevated levels of L-Dopa, therefore treatment with L-Dopa is counterintuitive, and only reported in 10 cases. However, in 4 patients with variants at the L-Dopa binding site, a sustained effect of L-Dopa was seen (see part I). Reported side effects (diarrhea and restlessness) in these patients were minimal and dose-dependent [19]. No improvement was reported in 3 cases [40, 42], and in 3 cases response was not described [2]. The use of carbidopa, an AADC inhibitor, is potentially dangerous in AADCD from a pathophysiological point of view because it further inhibits an already deficient enzyme. For dose recommendations of L-Dopa, see Table 4. In patients on L-Dopa therapy, extra attention should be given to folinic acid supplementation because of possible depletion.

**R#35 (strong):** L-Dopa is first line treatment **only** for patients with L-Dopa binding-site variants (e.g. p.[G102S], p.[R347Q], p.[R160W]). On theoretical grounds, L-Dopa without carbidopa is preferred.

**R#36 (conditional):** In patients without proven L-Dopa binding-site variants,, an L-Dopa trial can be considered if other treatment options fail.

**R#37 (strong):** CSF 5-MTHF levels should be determined before and during L-Dopa therapy.

#### Folinic acid

In theory, secondary cerebral folate deficiency may develop in AADCD since O-methylation of the excessive amounts of L-Dopa to 3-OMD depletes methyl donors including SAM and 5-MTHF. However, decreased levels of CSF 5-MTHF were only reported in a very limited number of patients (see part IIa). The therapeutic use of folinic acid in AADCD is reported in 4 patients [21, 22, 47]. In three patients, some improvement was seen. No side effects were published, but several members of the guideline working group reported gastro-intestinal side effects in AADCD patients (unpublished observations). Costs and insurance covering of folinic acid varies amongst countries. Low CSF 5-MTHF levels must be supplemented with folinic acid, not folic acid. Dose recommendations can be found in Table 4.

**R#38 (conditional):** Supplementation with folinic acid can be considered in all patients, and is clearly recommended when 5-MTHF in CSF is low.

**R#39 (research):** Further research on 5-MTHF is needed to be able to give strong recommendations on folinic acid supplementation and 5-MTHF follow-up in AADCD. At this moment, we do not recommend regular follow-up lumbar punctures in stable patients. In case of unexpected clinical deterioration, measurements of CSF 5-MTHF should be considered to rule out secondary folate deficiency.

#### 5-Hydroxytryptophan

5-HTP is the substrate for AADC to form serotonin, and its use in AADCD is as counterintuitive as the use of L-Dopa. Use of 5-HTP in AADCD was described in 5 patients [34, 40, 47, 56]. None showed a positive effect. In contrast, 2 patients showed side effects: lethargy, increased axial hypotonia, and abdominal pain.

**R#40 (strong):** Based on the current evidence and pathophysiological mechanisms, 5-HTP should not be used in the treatment of AADCD.

#### Selective serotonin reuptake inhibitors (SSRIs)

The use of SSRIs (paroxetine, ergotamine and fluoxetine) was reported in 4 patients with AADCD [5, 40, 45, 56]. Only one patient showed a possible, unspecified response. The other 3 patients showed no clinical benefit, but experienced side effects including worsening of oculogyric crises and hypotonia, lethargy, and dystonic reactions.

**R#41 (conditional):** Based on the current evidence, the use of SSRIs in AADCD is not recommended.

**Table 4:** Recommended drugs and doses in AADC deficiency

	Class	Drug	Mechanism	Dose recommendation	Precaution/comments
FIRST LINE TREATMENT AGENTS	Vitamin B6	Pyridoxine (vit B6)	Cofactor, optimizes residual AADC activity	Start: 100 mg/d in 2 doses Max 200 mg/d	May be preferred over pyridoxal 5-phosphate because of cost and availability Maintain for 1 year, then discontinue when in stable circumstances. If no deterioration, leave discontinued. Chronic use in high dose can cause severe sensorimotor polyneuropathy Side effects: generally well tolerated, sometimes nausea, vomiting.
		Pyridoxal 5-Phosphate	Cofactor, optimizes residual AADC activity	Start:100 mg/d in 2 doses. Max 200 mg/d	Consider trial if pyridoxine gives too many side effects or is not effective. Chronic use in high dose can cause severe sensorimotor polyneuropathy
	Dopamine agonists	Pramipexole	Non-ergot derived D2-agonist with preference for D3 receptor subtype.	Start 0.005 – 0.010 mg/kg/d of BASE in 1-3 divided doses, increase every 3-7* days by 0.005 mg/kg/d, max 0.075 mg/kg or 3.3 mg/d of BASE	Distinction in salt and base content. Take tablets with water, optional with food High risk of drug-induced dyskinesias
		Ropinirole	Non-ergot derived D2-agonist with preference for D3-receptor subtype.	Suggestion: Start 0.25 mg/d 1 daily 2 h before bedtime; increase every 3-7* days to 0.5-4.0 mg/d in 3 divided doses, max 0.3 mg/kg/d or 24 mg/d	Do not use in severe kidney failure Take tablets with food Very limited experience in AADC deficiency, physician should extrapolate and titrate carefully. Probably high risk of drug-induced dyskinesias as in other dopamine agonists.
		Rotigotine patch	Non-ergot derived D2 agonist with preference for D3; also effect on D2, D1 and D5; and α2B and 5HT1A agonist.	>12 years and >15 kg: Start 2 mg/d; weekly increase by 2 mg, max 8 mg/d.	No data available for use in children <12y/ <15 kg. Do not cut patches. Drug induced dyskinesias require a lower dose and/ or slower increase Skin reactions occur often (about 30 %). Sulphite can lead to allergic reactions Remove patch during MRI/ electrocardioversion (aluminium content)
		Bromocriptine	Ergot-derived D2-agonist with D1 receptor antagonist effect	Start 0.1 mg/kg/d (max 1.25 mg/d); increase weekly by 0.1 mg/kg/d (max 1.25 mg/d) up to 0.5 mg/kg/d (max 30 mg/d) in 2-3 divided doses.	Non-ergot derived dopamine agonists are preferred Take tablets with food Small risk of fibrotic complications, consider cardiac screening before and during use. Higher risk with higher dose, dose restricted to 30 mg/d in adults. Maintain lowest effective dose.
		*Pergolide or cabergoline*	*Ergot-derived*	*None*	Do not use because of higher risk of fibrotic complications
	MAO-inhibitors	Selegiline	MAO-B inhibitor (non-selective in very high doses)	Start 0.1 mg/kg/d in 2-3 divided doses. Increase every 2 weeks by 0.1 mg/kg/d up to 0.3 mg/kg/d or 10 mg/d	Dose at breakfast and lunch, avoid night-time doses if insomnia is experienced. Dose sublingual preparations much lower
		Tranylcypromine	Non-selective MAO-A and -B inhibitor	Start 0.1 mg/kg/d in 2 doses. Increase every 2 weeks by 0.1 mg/kg/d up to 0.5 mg/kg/d (max 30 mg)	Dose at breakfast and lunch, avoid night-time doses if insomnia is experienced. Occurrence of ‘cheese effect’ (hypertensive crises when foods with high content of tyramine are ingested) is very unlikely in patients with AADC deficiency due to their low levels of dopamine, norepinephrine and epinephrine.
ADDITIONAL SYMPTOMATIC TREATMENT	Anticholinergics (dystonia/ autonomic symptoms)	Trihexyphenidyl	Anticholinergic agents, restores neurotransmitter disbalance	<15 kg: start 0.5-1 mg/d in 1 dose; increase every 3-7* days by 1 mg/d in 2-4 doses/d >15 kg: start 2 mg/d in 2 doses; increase every 3-7* days by2mg/d in 2-4 divided doses. Effective dose highly variable (6-60 mg) Maximum dose: <10 kg 30 mg/d; >10 kg 60 mg/d	In general, the younger, the better tolerated; dosages often exceed recommended dose for adults (15 mg/d). Maximum dose is dictated by side effects: e.g. dry mouth, dry eyes, blurred vision (mydriasis), urine retention, constipation. Sedation in high doses.
		Benztropine	Centrally acting anticholinergic agent. Also dopaminergic effect by inhibiting presynaptic reuptake	Start 1 mg in 2 divided doses, increase weekly up to 4 mg/d	Anticholinergic side effects: e.g. dry mouth, dry eyes, blurred vision (mydriasis), urine retention, obstipation. Sedation in high doses.
	Nasal congestion	Oxymetazoline or xylometazoline nosedrops	α-adrenergic agonist leading to local vasoconstriction	Use general dose guidelines for age, try to use lowest available dose in chronic use	Try to include intermittent weeks without treatment to prevent habituation Hypertensive crises if used concomitantly with MAO-inhibitors is very unlikely in patients with AADC deficiency due to their levels of dopamine, norepinephrine and epinephrine
	Sleeping problems	Melatonin	Regulates onset of sleep and day/night cycling	Start 3 mg/d, given 4 h before onset of sleep. Max dose 5-8 mg/d	Transient night terrors on initial treatment can occur (personal experience) Availability differs between countries.
	Irritability/ sleep disturbance	Clonidine	Centrally acting antihypertensive drug; imidazoline (I1-) and α2-agonist	Start 0.1 mg/d ante noctum, increase to max 3 mg/d ante noctum	Monitor blood pressure in higher dose Sedative, therefore give AN
SPECIAL CASES ONLY	L-Dopa binding site variant	L-Dopa without carbidopa	Substrate for AADC to form dopamine; effective in certain binding site variants	Start 0.5-1 mg/kg/d in 3 divided doses, increase 2 weekly by 1 mg/kg to 5 mg/kg/d. Only if clinical effective, further increase to max 15 mg/kg/d	Start as first line treatment only if known binding site variant. Otherwise, consider as third-line treatment trial for 2 months (or less if deterioration) when in stable clinical situation. Monitor CSF during treatment, including 5-MTHF
	Low 5-MTHF in CSF	Folinic acid (calcium folinate)	Methylation of excessive amounts of L-Dopa in AADCD may cause depletion of methyl donors.	1-2 mg/kg/d, max 20 mg/ d	Only supply if 5-MTHF is low in CSF Monitor 5-MTHF in CSF during treatment with L-Dopa

### Treatment schedule AADCD

**Fig. 2:**
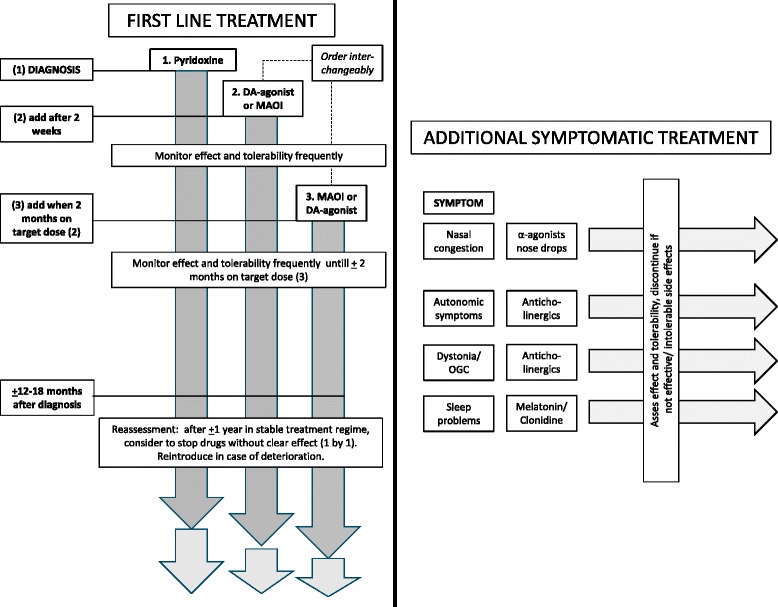
Treatment algorithm in AADC deficiency. This figure reflects a possible treatment scheme for a newly diagnosed AADCD patient. Drug dose and escalating schemes are given in Table 4. At the left, first line treatment is depicted in which pyridoxine is started at diagnosis (step (1)), followed after two weeks by step (2): either one of the dopamine agonists in escalating scheme or one of the MAO-inhibitors. After two months of treatment at target dose, step (3) is added: either a dopamine agonist or a MAO-inhibitor. The order of introducing dopamine agonist or MAO-inhibitors is interchangeably. Dose escalation depends on effect and tolerability. If an agent is not effective or has too many side effects, consider replacing it with another agent from the same class before going to the next step. In case of intolerable side effects, treatment should be stopped. After about 1 year in stable treatment regimen, reassessment should take place: consider to discontinue drugs (only 1 at a time) without clear treatment effect. Frequent assessment is then again necessary, and agents should be reinstated in case of deterioration. At the right, additional symptomatic treatment is depicted, with different drug classes that could be added for specific symptoms. Always avoid starting more than 1 agent at the time. Assess tolerability and effect frequently, and discontinue drugs that have no clear effect, or give intolerable side effects. Treatment in special situations (L-Dopa, folinic acid) is not depicted in this figure (see text). Abbreviations: DA-agonist: dopamine agonist; MAOI: MAO-inhibitor; OGC: oculogyric crisis

In Table 4, recommended drugs and doses are summarized. Figure 2 shows a schematic example of how a patient with newly diagnosed AADCD can be treated. In recommendation 42 the most important treatment principles of AADCD are given:

**R#42 (strong):** The core recommendations for medical treatment of AADCD are:

1. First line treatment agents are selective dopamine agonists, MAO-inhibitors, and pyridoxine
2. Additional symptomatic treatment agents are anticholinergic agents, melatonin, benzodiazepines, and alpha-adrenoreceptor blockers.
3. In general, therapy with multiple drugs will be needed and doses should be titrated individually and sequentially.
4. General treatment principles to adhere to are: step-wise approach, start low and go slow when increasing the doses, and discontinue/ wean off medication that is not helpful.

**R#43 (research):** Since the evidence for medical treatment in AADCD is low to very low, it would be desirable to conduct randomized clinical trials in AADCD to improve the proposed treatment schedule.

### Drugs to avoid in AADCD

Centrally acting dopamine antagonists, used for their antiemetic and antipsychotic properties, should be avoided in AADCD because they have the potential to worsen symptoms of dopamine deficiency. The detrimental effects of haloperidol (antagonist at dopamine 2 receptors) are illustrated in one case report [39]. Metoclopramide should not be used for the treatment of nausea. Levomepromazine, an agent with antagonistic properties to epinephrine, histamine, acetylcholine, dopamine, and serotonin, led to severe side effects in one patient [46]. Less is known about serotonin antagonists like 5-HT3 receptor blockers that are used as antiemetics (e.g. ondansetron, granisetron). From a pathophysiological standpoint, side effects can be expected and their use should be avoided, but there is no literature or clinical experience available. It is important to realize that many drugs have antagonistic properties for several neurotransmitters, and before introducing any drug to AADCD patients, its potential benefits and harms should be considered carefully.

In case of nausea and vomiting in AADCD patients, supportive care to avoid dehydration and hypoglycemia is most important. If possible, anti-dopaminergic and anti-serotonergic agents should be avoided. If medical therapy is needed, low dose domperidone can be considered. Local guidelines on availability, cardiac concerns and dose recommendations should be followed. Although domperidone is a dopamine antagonist, it does not cross the blood brain barrier and therefore side-effects in AADCD are expected to be limited.

**R#44 (strong):** Centrally acting dopamine antagonists should be avoided in AADCD.

**R#45 (GPP):** Supportive care in nausea and vomiting in AADCD patients should be optimal. If medical treatment is needed, low-dose domperidone can be considered.

### Dystonic crisis (status dystonicus)

Dystonic crisis can occur in AADCD patients, but limited data is available about the prevalence of and treatment approach to this rare, potentially life-threatening complication. A dystonic crisis is often triggered by infection or medication adjustments. It is characterized by sustained severe dystonic muscle contractions that can lead to airway compromise and metabolic complications such as rhabdomyolysis leading to acute renal failure [66]. The approach to dystonic crisis in AADCD should be similar to the general approach, with admission to an intensive care unit, fluid and nutrition supplementation, sedation (usually with benzodiazepines) and respiratory support if needed. Dopamine antagonists and L-Dopa should not be used in AADCD. For a review, see [67].

**R#46 (GPP):** Dystonic crisis in AADCD is a potentially life-threatening condition and should be treated promptly.

### IIIb: Non-medical treatment

#### Paramedical treatment in AADCD

Although there are no studies or reports on the effects of paramedical treatment in AADCD, a multidisciplinary approach with physiotherapy, speech therapy, occupational therapy, feeding and nutritional assessment, and (neuro)psychological treatment and support is essential to prevent secondary complications and promote development. A physiatrist (rehabilitation specialist) is also a valuable member of the team. In this respect, approach to a patient with AADCD is comparable to the approach of other chronic neurological disorders, e.g. cerebral palsy.

**R#47 (GPP):** Involvement of a multidisciplinary team that includes a rehabilitation physician (physiatrist) and allied health professionals (paramedical therapy services) is essential in the care for AADCD patients.

#### Gene therapy and other surgical treatment options

Gene therapy in which a viral vector (adeno-associated virus type 2) encoding cDNA of the human *DDC* gene is delivered to targeted brain structures was originally developed for patients with Parkinson’s disease [68]. Gene transfer to the bilateral putamen has been performed in four AADCD patients in Taiwan with modest but promising results [24]. Further research is ongoing. Clinical trials are presently recruiting patients in Taiwan and Japan [11, 12], and trials in the USA and Europe, in which gene transfer will target midbrain structures, are anticipated [69]. There is no experience with deep brain stimulation or other surgical treatment options in AADCD.

**R#48 (research):** Gene therapy for AADCD is currently under development in research setting. Clinical trial results will determine whether further implementation of this promising therapy may occur in the future.

## Part IV: Complications and long-term management in AADCD

### Cardiac decompensation

Structural cardiac abnormalities in AADCD are not expected from a pathophysiological perspective. In one case report, electrocardiogram and heart ultrasound were reported as normal [34], but in most reports information regarding cardiac status is lacking. There are individual descriptions of one patient with bradycardia on ECG [20], and one patient who suffered from a witnessed cardiac arrest at age 9 years, with ensuing permanent neurological damage [59]. It is possible that cathecholaminergic deficiency and autonomic dysfunction may potentially lead to cardiac complications, which may be more evident during illness or stress (e.g. intercurrent infections, surgical interventions).

**R#49 (strong):** There should be awareness for possible cardiac complications in AADCD patients during potentially stressful situations and cardiac monitoring during these instances is recommended. Regular follow-up cardiac screening is not needed, but recommended before any anesthesia or intervention. If available in local care setting, cardiac screening should consist of a referral to a (pediatric) cardiologist for clinical evaluation, ECG and echocardiogram. For cardiac screening for patients on dopamine agonists, see recommendation #28.

### Orthopaedic complications

Orthopaedic complications are reported in AADCD secondary to severe motor impairment (abnormalities of tone, posture, paucity of movement). Contractures leading to mobilization problems are described in a few patients [7, 39]. A multidisciplinary approach for follow-up and treatment is recommended. Orthopaedic monitoring (e.g. hip and spine X-rays) is recommended, preferably in a local care setting.

**R#50 (GPP):** AADCD patients should be monitored for orthopaedic complications following standards of good care for patients with motor impairment.

### Infections

There is an increased risk of infections in AADCD patients, likely not because of disease-specific mechanisms but secondary to complications including impaired feeding and swallowing, reduced mobility, and recurrent hospitalization. A disease specific aspect of AADCD could be the reduced response to stress of infections, which can be fatal. Treating physicians (specialists and primary care physicians) should be aware of this. Furthermore, hyperthermia in AADCD can be due to autonomic temperature dysregulation and may not always represent a fever due to underlying infection. Vaccinations following local vaccination programs are recommended.

**R #51 (GPP):** Because AADCD patients have a reduced response to stress, close monitoring during infections is recommended.

**R#52 (GPP):** Vaccinations following local vaccination programs are recommended in AADCD.

### Follow-up visits

**R#53 (strong):** AADCD patients should be seen at least yearly by a (child) neurologist with experience in movement disorders or neurometabolic disease, ideally in a multidisciplinary setting (see R#46), facilitating a shared-care approach.

### Emergency card

**R#54 (GPP):** It is recommended that all AADCD patients receive an emergency card including short information on AADCD, possible complications, and drugs to avoid in this disorder. An agreed emergency card can be found as Additional file **3**.

## Part V: Social issues and transition

### Psychological support for families

There are no reports on the health and well-being of caregivers of children with AADCD available. However, studies in families with children with chronic diseases show that caregivers are at risk of reduced quality of life. Family stress and parental coping are factors that are associated with quality of life [70]. For more background information and strategies to improve support for parents of chronically ill children, see e.g. Newton and Lemarche [71]. Quality of life assessment and the evaluation of disease burden in family members of AADCD patients are an essential part of the ongoing iNTD registry study [9].

In isolated case reports, an increased prevalence of psychiatric disorders in carriers of AADCD has been described. To date, this has neither been systematically evaluated nor consistently reported [35, 45].

**R#55 (strong):** If available in local care setting, psychological support (e.g. psychologist, social worker) to caregivers, siblings, and patients with AADCD is recommended

**R#56 (research):** Further research is needed to clarify whether *DDC* gene variant carriers have a primary (genetically determined) increased risk of psychiatric symptoms.

### Genetic counseling

Although there is no specific literature on genetic counseling in AADCD available, it can be considered good clinical practice for all parents of children with AADCD to be offered standard genetic counseling, considering the autosomal recessive nature of the disorder. See also R#60.

**R#57 (strong):** If available in local care settings, all parents of patients with AADCD should be offered standard genetic counseling.

### Parental organizations

There are two internationally operating non-profit parental organizations for patients with AADCD: the UK based AADC Research Trust (www.aadcresearch.org) and the USA based Pediatric Neurotransmitter Disease Association (www.pndassoc.org). Furthermore, there is the Spanish neurotransmitter diseases association “De neu” (www.deneu.org). The major aim of these organizations is to help and support patients with AADCD by providing information and organizing family meetings, promoting disease awareness and funding scientific research.

**R#58 (strong):** It is recommended that caregivers of AADC deficient patients be informed about existing parental organizations for this rare disorder.

### Transition from child to adulthood

No specific reports are available on the healthcare transition of patients with AADCD from childhood to adolescence to adulthood. Most reports focus on patients in infancy and childhood. There is no information published on puberty. Also fertility concerns or pregnancy has not been described in AADCD. Considering the often severe phenotype, for the majority of patients this will not be an issue. However, relatively mildly affected patients have been described [35] and the phenotypic spectrum continues to expand. Based on experience with other disorders that cause neurodevelopmental disability, it is known that there are significant gaps in our knowledge and practice about healthcare transition for young adults [72]. The transfer from intense, family-oriented pediatric care, to primarily individually focused adult care is often perceived as difficult for both patients and caregivers [73]. Local differences in health care organization for patients with neurodevelopmental disabilities will be present across countries. Based on the literature, it is not possible to give specific recommendations on transition of care for patients with AADCD. However, the guideline committee clearly states that this process should be adequately prepared. Continuation of the multidisciplinary care setting is recommended.

**R#59 (GPP):** Transition of AADCD patients to adult care should be prepared well, and continue to take place in specialized centers. Multidisciplinary care should be continued.

**R#60 (research):** Follow-up studies of adult patients with AADCD are strongly recommended to better define prognosis and possible new clinical symptoms and signs in adolescence and adulthood.

## Part VI: Special situations

### Anesthesia for interventions

It is not unusual for patients with AADCD to need anesthesia for (minor) interventions such as gastrostomy. Only two reports described anesthesia management in two different AADCD patients [29, 30]. It should be noted that intravenous phenylephrine and dopamine may lead to an exaggerated hemodynamic response in these patients. In case of hypotension during intervention, dopamine should be initiated at low dose (e.g. 1-2 ug/kg/min) [29]. Monitoring of hemodynamic function, temperature and glucose levels is important, especially in prolonged interventions.

**R#61 (strong):** Anesthesia should be well prepared in patients with AADCD because patients have an increased risk of hemodynamic instability and hypoglycemia. In addition to cardiovascular monitoring, monitoring of temperature and glucose levels during procedures is recommended. In general, it is recommended to continue standard treatment (including MAO inhibitors) during interventions.

### Intensive Care management

Information on ICU management of AADC deficient patients is limited. In a case report of one patient, cardiac complications are described during ICU treatment with exogenous catecholamines [39]. Another case report described one patient with a 5-h period of hypotension with bradycardia following sedation at age 11 years [59]. Autonomic testing in two patients showed severe impairment of sympathetic blood pressure modulation in both. In the more severely affected patient, recurrent episodes of cardiorespiratory arrest, preceded by profound bradycardia, were described, evoked by painful stimuli (e.g. blood draw) [45].

**R#62 (strong):** AADCD leads to sympathetic impairment with possible cardiac complications. This should be anticipated during ICU admissions.

### Prenatal diagnosis

Prenatal diagnosis of AADCD might be possible by measuring 3-OMD, 5-HTP and L-Dopa in amniotic fluid or fetal plasma [74], but if both disease-causing genetic variants are known, the most reliable method of prenatal diagnosis is to perform genetic analysis of chorionic villi or amniotic fluid cells.

In two reports, successful pre-implantation genetic diagnosis has been described. In four of five families, unaffected live births were achieved [75]. In a report from the same group, Chen et al describe this technique in a diverse group of single gene disorders including AADCD [76].

**R#63 (strong):** If local resources allow, prenatal genetic diagnosis or preimplantation techniques should be offered to carriers of AADCD.

## Conclusions

AADCD is an early onset disorder with a combined deficiency of serotonin, dopamine, norepinephrine and epinephrine. Key clinical symptoms are hypotonia, movement disorders (especially oculogyric crisis, dystonia and hypokinesia), developmental delay, and autonomic symptoms. The phenotypic spectrum is broad and can range from very severe to relatively mild. There are no clear genotype/ phenotype correlations in AADCD except for the homozygous IVS6 + 4A > T splice variant that is associated with a severe phenotype in all cases reported to date, and has an increased incidence in people of Chinese ethnicity.

There are three core diagnostic tools for identifying AADCD:

1. Low CSF levels of 5-HIAA, HVA and MHPG, with normal CSF pterins, and increased CSF levels of L-Dopa, 3-OMD and 5-HTP
2. Genetic diagnosis showing compoung heterozygous or homozygous disease causing variants in the *DDC* gene
3. Decreased AADC enzyme activity in plasma

To diagnose AADCD, genetic testing should be performed and at least two out of three core diagnostic tests should be positive. If local resources allow, we recommend performing all three key diagnostic tests in patients with this rare disorder.

The core recommendations for treatment of AADCD are:

1. First line treatment with selective dopamine agonists, MAO-inhibitors, and pyridoxine;
2. Additional symptomatic treatment agents with anticholinergic agents, melatonin, benzodiazepines, and alpha-adrenoreceptor blockers
3. In general, multiple drug classes will be needed
4. Overall treatment principles to adhere to are: step-wise approach, start low and go slow when increasing dosages, and discontinue/ gradually withdraw medication that is not helpful.

Involvement of a multidisciplinary team, including physiatrist and paramedical therapy services, is essential in the care for AADCD patients, and psychological support should be offered to caregivers, siblings and patients. Patients and caregivers should be informed about the AADCD parental organizations. AADCD patients should be seen at least yearly by a (child) neurologist with experience in movement disorders or neurometabolic disease, ideally in a multidisciplinary setting, facilitating a shared-care approach.

Although the level of evidence mainly consisted of level 3 and 4 (non-analytical studies and expert opinion), using SIGN and GRADE methodology permitted us to form the strong and conditional recommendations provided in this guideline. We believe that this consensus guideline will improve the care of AADCD patients around the world. Furthermore, we determined several knowledge voids that should be addressed in future research.

## Additional files

Additional file 1: List of Key Questions. (DOCX 28 kb)
Additional file 2:
**Figure S1.** Flow chart showing the systematic literature search and number and type of included sources. (PPTX 81 kb)
Additional file 3: AADCD Emergency card. (DOCX 16 kb)

## Abbreviations

3-OMD: 3-O-Methyldopa
5-HIAA: 5-Hydroxyindoleacetic acid
5-HTP: 5-Hydroxytryptophan
5-MTHF: 5-Methyltetrahydrofolate
AADC: Aromatic l-amino acid decarboxylase
AADCD: Aromatic l-amino acid decarboxylase deficiency
COMT: Catechol O-methyl transferase
CSF: Cerebrospinal fluid
DBS: Dried blood spot
EEG: Electroencephalography
GPP: Good practice point
HVA: Homovanillic acid
iNTD: International Working Group on Neurotransmitter Related Disorders
MAO: Monoamine oxidase
MHPG: 3-Methoxy-4-hydroxyphenylglycol
PLP: Pyridoxal phosphate
PNPO: Pyridox(am)ine 5-phosphate
SAM: s-Adenosylmethionine
SIGN: Scottish Intercollegiate Guideline Network
SSRI: Selective serotonin reuptake inhibitors
VLA: Vanillactic acid
VMA: Vanillylmandelic acid
