# Supplementary material for: Consensus guideline for the diagnosis and treatment of aromatic l-amino acid decarboxylase (AADC) deficiency
Source: Orphanet J Rare Dis. 2017 Jan 18;12:12. doi: 10.1186/s13023-016-0522-z (PMC5241937; doi:10.1186/s13023-016-0522-z)
Supplement: Additional file 1: — List of Key Questions. (DOCX 28 kb) [file 13023_2016_522_MOESM1_ESM.docx]

**Additional file 1: List of Key Questions per working group**

**Part I: Clinical Presentation (working group 1):**

1.1: For each component in **Table 1**, describe:

1.1.1: which signs and symptoms are described in AADCD before treatment?

1.1.2: which signs and symptoms are described in AADCD during follow-up (in course of the

disease, not as adverse treatment effects)?

1.1.3: what is age of onset of each symptom/ sign?

1.2: what is age of diagnosis of reported patients with AADCD?

1.3: what is level of functioning in AADCD?

1.3.1: what developmental milestones are reached before treatment?

1.3.2: what developmental milestones are reached during treatment?

1.3.3: is there loss of skills in untreated AADCD patients?

1.3.4: is there loss of skills in treated AADCD patients?

1.4: Genotype/ biochemical phenotype/ clinical phenotype correlations (focus on severity/ level of functioning were possible):

1.4.1: is there a genotype/ phenotype correlation in AADCD regarding symptomatology?

1.4.2: is there a correlation between values of CSF neurotransmitter metabolites and

phenotype?

1.4.3: is there a correlation between values of AADC-activity assay in plasma and phenotype?

**Table 1: symptoms and signs in clinical presentation of AADCD**

| A | Neurological disorders | | |
| --- | --- | --- | --- |
|  | A.1 | Movement disorders | Give a detailed description of each disorder:   - Head control - Hypokinesia - Voluntary movements - Hypotonia - Hypertonia - Dystonia - Dyskinesia - ... |
|  | A.2 | Eye movement disorders | - Oculogyric crisis - ... |
|  | A.3 | Speech development | - Dysartrhria - Language development |
|  | A.4 | Cognitive development | Describe |
|  | A.5 | Behavioural problems | Describe |
|  | A.6 | Developmental milestones | - Motor (describe) - ... |
|  | A.7 | Sleeping disturbances | - Hypersomnia - Insomnia - Fatigability - Diurnal fluctuations - ... |
|  | A.8 | Autonomic disorders | - Ptosis - Temperature instability - Sweating - Drooling (including hypersalivation) - Nasal congestion - ... |
|  | A.9 | Epilepsy | Describe |
|  | A.10 | Other | Include any other reported neurological symptom/ sign |
| B | Non- neurological disorders | | |
|  | B.1 | Cardiovascular disorders | - Bradycardia - Hypotension - ... |
|  | B.2 | Upper-airway disorders | - Laryngomalacia - ... |
|  | B.3 | Gastro-intestinal disorders | - Obstipation - Diarrhoea - Feeding problems - Tube feeding - ... |
|  | B.4 | Metabolic disorders | - Hypoglycemia - Renal function - Electrolyte disturbances - ... |
|  | B.5 | Endocrine disorders | - Growth Hormone - Thyroid function - .... |
|  | B.6 | Other | Include any other reported non-neurological symptom/ sign |

**Part IIa: Diagnosis, Laboratory Tests (Working Group 2)**

2.1 Describe the following available diagnostic tests:

2.1.1 Lumbar puncture

2.1.1.1 Describe for each of the measurements in **table 2:**

a. Diagnostic value

b. Recommened method and handling

c. Centers where this test can be performed

d. Reference value

e. Medication that should be stopped before initial diagnostic test (if any)

**Table 2: CSF measurements in AADCD**

| 1 | Neurotransmitter metabolites (HVA/5-HIAA/5-HTP/3-OMD, other) |
| --- | --- |
| 2 | Pterines |
| 3 | Folate |
| 4 | Vitamine B6/ PLP/ Pyridoxal phosphate |
| 5 | Other (describe…) |

2.1.2 AADC-activity measurement in plasma

a. Diagnostic value

b. Recommended method and handling

c. Centers where this test can be performed

d. Reference value

e. Medication that should be stopped before initial diagnostic test (if any)

2.1.3 Genetic Diagnosis:

2.1.3.1. Gene analysis of *DDC:*

a. Diagnostic value

b. Recommened method and handling

c. Centers where this test can be performed

d. List of mutations

2.1.3.2. Panel diagnostics:

a. Diagnostic value, what is known?

2.1.3.3. Whole exome sequencing

a. Diagnostic value, what is known?

2.1.4. Blood tests:

2.1.4.1. Prolactine

a. Diagnostic value

b. Reference values

c. Can this test be used to evaluate treatment response? (follow-up)

2.1.4.2. Melatonin

a. Diagnostic value

b. Reference values

c. Can this test be used to evaluate treatment response? (follow-up)

2.1.4.3. Other

a. Describe

2.1.5 Urine Tests

2.1.5.1. Vanillactic acid

a. Diagnostic value

2.1.5.2. Other

b. Describe

2.1.6 Other tests (e.g. saliva)

a. Describe

2.1.7. is there any evidence for newborn screening tests?

2.1.7.1. 3-OMD?

2.1.7.2. Other?

**Part IIb: Diagnosis; imaging and electroencephalography (working group 1)**

2.1.8 Radiological examinations

2.1.8.1. Magnetic resonance imaging (MRI) of the brain

a. What abnormalities are described?

b. What is the diagnostic value?

2.1.8.2. Nuclear imaging

a. What techniques are described?

b. What abnormalities are described?

c. What is diagnostic values?

2.1.9. Electroencephalography

a. What abnormalities are described?

b. What is the diagnostic value?

**Diagnosis, general:**

- What are mandatory tests for definite diagnosis of AADCD?

**Part III: Treatment (Working Group 3)**

Part IIIa: medical treatment of AADCD

3.1. Describe for all of the following possible drug options for chronic/ maintenance drugs in

AADCD in **table 3:**

1. Evidence: describe effect outcome specifics (for outcomes, see Table 1)
2. Dosage
3. Effects
4. Side Effects
5. Subset of patients for which this drug has better or worse outcome (if any)

**Table 3: list of different drugs used in maintenance therapy of AADCD**

| **NO** | **Class** | **Specific drugs** |
| --- | --- | --- |
| 1 | Dopamine Agonists | Bromocriptine |
|  |  | Pramipexol |
|  |  | Ropinirol |
|  |  | Rotigotine patches |
|  |  | Pergolide |
|  |  | Cabergolide |
| 2 | Levodopa | na |
| 3 | Levodopa/ Carbidopa | na |
| 4 | 5-Hydroxytryptophan | na |
| 5 | MAO-inhibitors | Selegiline |
|  |  | Trancylpromine |
|  |  | Other |
| 6 | Pyridoxine (B6) | na |
| 7 | Pyridoxal Phosphate | Na |
| 8 | Anti-cholinergic agents | Trihexyphenidyl |
|  |  | Biperiden |
|  |  | Others |
| 9 | Folate | na |
| 10 | Melatonin | na |
| 11 | Selective Serotonin Reuptake Inhibitors | Describe |
| 12 | Anti-epileptic drugs | Gabapentine |
| 13 | Benzodiazepines | Describe |
| 14 | COMT-inhibitors | Describe |
| 15 | Alfa-adrenoreceptor agonists | Describe |
| 16 | Benzodiazepines | Describe |
| 17 | Others | Describe |

**Part IIIb: non-medical treatment in AADCD**

3.2 What paramedical therapy is recommended in AADCD?

*If no evidence is available specific for AADCD, search for evidene for children with chronic*

*neurological disorders in general, e.g. cerebral palsy)*

3.2.1. Physiotherapy

3.2.2. Speech therapy

3.2.3. Dietary

3.2.4. Occupational therapy

3.2.5 (Neuro)Psychological therapy

3.3 What is the evidence for surgical treatment options in AADCD?

3.3.1. Deep brain stimulation

3.3.2. Gene Therapy

3.4 Contra-indications

3.4.1. What drugs are to be avoided in AADCD?

3.4.1.1. Neuroleptics

3.4.1.2. Other…

3.5 What drugs can be used in acute settings, e.g.g for treatment of dystonic crisis?

a. Described dosage, effect, and evidence

3.5.1. Baclofen

3.5.3 Benzodiazepines

3.5.4. Chloralhydrate

3.5.5. Phenobarbitone

3.5.6. Cannabinoids

3.5.7. Clonidine

3.5.8. Other

**Treatment, general:**

- What is the proposed algorithm for maintenance therapy of AADCD?

**Part IV: Complications and long-term management of AADCD patients (working group 4)**

4.1. Long-term complications:

4.1.1 Describe for each of the following complications their prevalence, proposed

treatment and proposed monitoring:

4.1.1.1. Cardiac decompensation

4.1.1.2. Orthopaedic complications (e.g. contractures, luxations)

4.1.1.3. Infections

4.1.1.4. Other long-term complications

4.2. Follow-up visits

4.2.1. Which topics should be covered on follow-up visits?

4.2.2. What monitoring tests are recommended and how should they be performed?

4.2.2.1. Nutritional monitoring

4.2.2.2. Orthopaedic monitoring

4.2.2.3. Cardic monitoring

4.2.2.4. Lab tests (if any)

4.2.2.5. Specific biochemical monitoring (lumbar puncture?)

4.2.2.6. Radiological monitoring

4.2.2.7. Cognitive monitoring

4.2.2.8. Other

**Long-term monitoring, general:**

- What is the proposed long-term management approach for AADCD?

**Part V: Social issues and transition (working group 5)**

What is known about, or can be recommend, regarding:

5.1. Psychological support for families

5.2. Genetic counseling

5.3. Parental organizations

5.3.1. Contact information

5.4. Transition to adulthood

5.5. Fertility issues/ pregnancy

**Part VI: Special situations (working group 6)**

Describe what is known of, and recommended care in:

6.1. Anesthesia for interventions

6.2. Intensive care management

6.3. Prenatal diagnosis
