# Supplementary material for: Consensus guideline for the diagnosis and treatment of aromatic l-amino acid decarboxylase (AADC) deficiency
Source: Orphanet J Rare Dis. 2017 Jan 18;12:12. doi: 10.1186/s13023-016-0522-z (PMC5241937; doi:10.1186/s13023-016-0522-z)
Supplement: Additional file 2: — Figure S1. Flow chart showing the systematic literature search and number and type of included sources. (PPTX 81 kb) [file 13023_2016_522_MOESM2_ESM.pptx]

## Slide 1
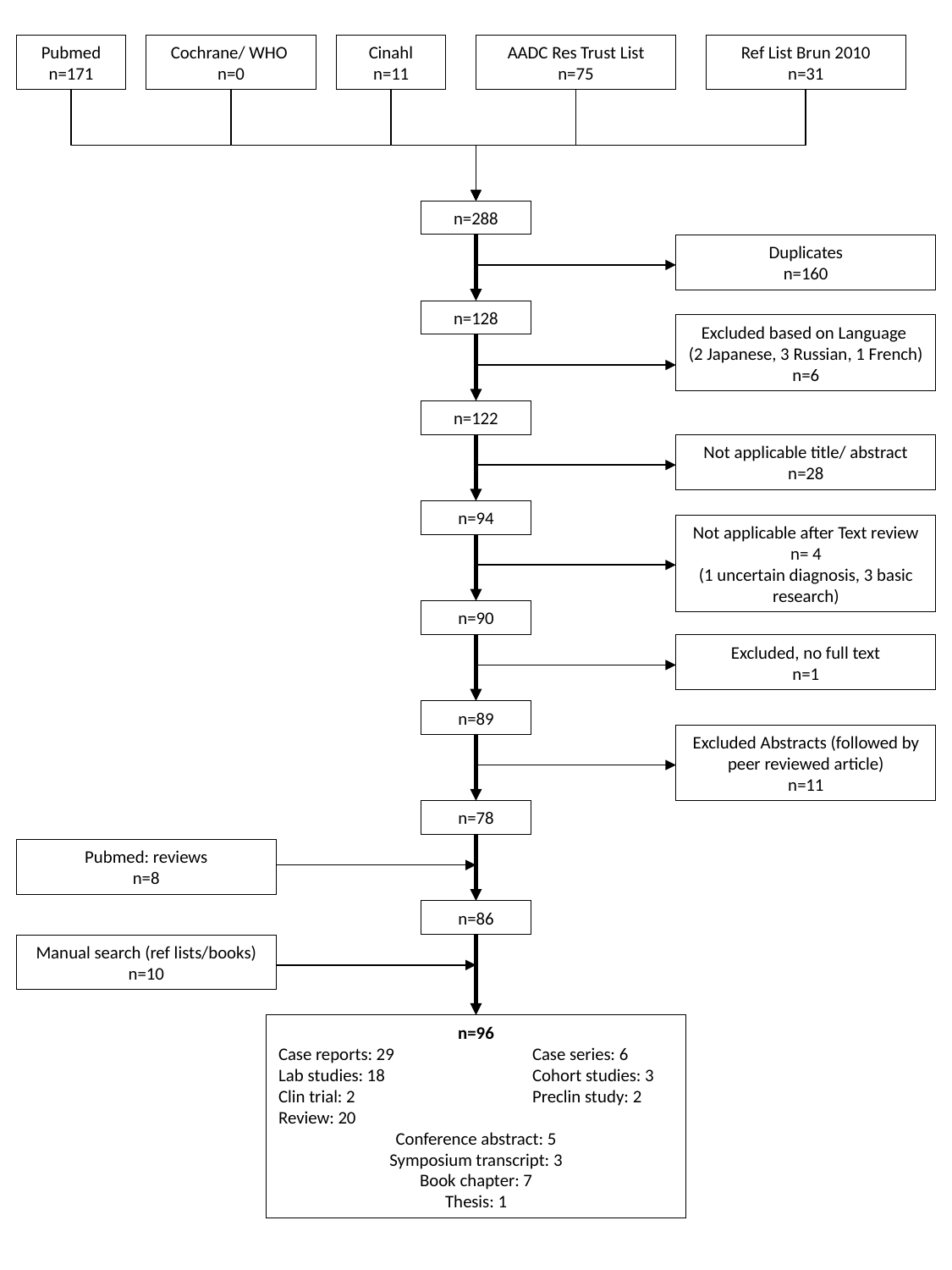

Pubmed
n=171
Cochrane/ WHO
n=0
Cinahl
n=11
AADC Res Trust List
n=75
Ref List Brun 2010
n=31
n=288
Duplicates
n=160
n=128
Excluded based on Language
(2 Japanese, 3 Russian, 1 French)
n=6
n=122
Not applicable title/ abstract
n=28
n=94
Not applicable after Text review
n= 4
(1 uncertain diagnosis, 3 basic research)
n=90
Excluded, no full text
n=1
n=89
Excluded Abstracts (followed by peer reviewed article)
n=11
n=78
Pubmed: reviews
n=8
n=86
Manual search (ref lists/books)
n=10
n=96
Case reports: 29		Case series: 6
Lab studies: 18		Cohort studies: 3
Clin trial: 2 		Preclin study: 2
Review: 20
Conference abstract: 5
Symposium transcript: 3
Book chapter: 7
Thesis: 1
