# Supplementary material for: Consensus guideline for the diagnosis and treatment of aromatic l-amino acid decarboxylase (AADC) deficiency
Source: Orphanet J Rare Dis. 2017 Jan 18;12:12. doi: 10.1186/s13023-016-0522-z (PMC5241937; doi:10.1186/s13023-016-0522-z)
Supplement: Additional file 3: — AADCD Emergency card. (DOCX 16 kb) [file 13023_2016_522_MOESM3_ESM.docx]

**Additional file 3: Emergency Card**

Aromatic L-amino acid decarboxylase deficiency

**Synonyms:**

AADC-deficiency

AADCD

**Definition**

AADC-deficiency is a rare autosomal recessive neurometabolic disorder that leads to a deficiency of the neurotransmitters serotonin, dopamine, norepinephrine (noradrenaline) and epinephrine (adrenaline).

**Key clinical symptoms:**

- Severe hypotonia
- Movement disorders
  - Oculogyric crisis, dystonia, hypokinesia
  - **Prolonged oculogyric crisis/ status dystonicus possible**
- Developmental delay
- Autonomic symptoms
  - Excessive sweating, drooling, temperature instability, hypotension
  - Sympathetic impairment with possible cardiac complications
- **Impaired stress response**
  - **Hypoglycemia possible**
  - **Sudden death possible**

**Emergency treatment:**

- Clinical admission and surveillance, including monitoring of glucose levels, during acute illness and/ or any medical intervention

**Attention:**

- Anesthesia should be well prepared because of increased risk of hemodynamic instability and hypoglycemia. Ensure regular monitoring of temperature and glucose levels. Standard treatment (including MAO inhibitors) should be continued before and during interventions.

**Drugs to avoid:**

- DO NOT GIVE any centrally acting **antidopaminergic medication** (e.g. metoclopramide, haloperidol and most other neuroleptics)
- Avoid **phenothiazines** because of antidopaminergic, antiadrenergic and antiserotonergic properties
- Caution when using drugs with **antiserotonergic properties** (e.g. ondansetron)
- Low dose of domperidone can be considered if antiemetic treatment is needed.
